# Uncovering the characteristics of air pollutants emission in industrial parks and analyzing emission reduction potential: case studies in Henan, China

**DOI:** 10.1038/s41598-021-03193-z

**Published:** 2021-12-09

**Authors:** Gengyu Gao, Shanshan Wang, Ruoyu Xue, Donghui Liu, He Ren, Ruiqin Zhang

**Affiliations:** 1 College of Chemistry, Zhengzhou University, Zhengzhou, 450001 China; 2 School of Ecology and Environment, Zhengzhou University, Zhengzhou, 450001 China

**Keywords:** Environmental impact, Atmospheric science

## Abstract

Industrial parks contribute greatly to China’s economic development while emitting huge air pollutants. It is necessary to study the characteristics of air pollutant emissions in industrial parks. In this study, emission inventories for 11 industrial parks were established. Meanwhile, the source emission and spatial distribution characteristics of the industrial park were analyzed. The cluster analysis was used to classify these parks into “4Hs”, “Mixed” and “4Ls” parks. “4Hs”, “Mixed” and “4Ls” represent that the levels of energy intensity, economic proportion of energy-intensive industries, coal proportion and pollution performance value are high, medium and low in turn. Then three emission reduction measures were set up to estimate the emission reduction potential and environmental impacts. The results show that: (1) the emissions of SO_2_, NO_x_, CO, PM_10_, PM_2.5_, VOCs and NH_3_ of 11 industrial parks in 2017 were 11.2, 23.1, 30.8, 8.3, 3.5, 5.1, and 1.1 kt, respectively. (2) Power plants were the largest source of SO_2_ and NO_x_ emissions, and industrial processes were the largest emission source of CO, PM_10_, PM_2.5_, VOCs and NH_3_. (3) “4Hs” parks with traditional energy-intensive industries as the leading industries should be the emphasis of air pollutant emission reduction. (4) Through the optimal emission reduction measures, SO_2_, NO_x_, PM_10_, PM_2.5_ and VOCs were reduced by 81, 46, 51, 46 and 77%, respectively. Environmental impact reductions include 1.6 kt SO_2_eq acidified gas emissions, 1.4 kt PO_4_^3−^eq eutrophication substances, 4.2 kt PM_10_eq atmospheric particulate emissions, 7.0 kt 1,4-DCEeq human toxic substances, and 5.2 kt PM_2.5_ eq breathing Inorganic. This study is helpful to understand the characteristics of air pollutants emissions in industrial parks and promotes the proposal and implementation of air pollutant emissions reduction strategies.

## Introduction

An industrial park is an industrial cluster designed to meet the compatible needs of different organizations in the same area^1^. China began to attach importance to the establishment and development of industrial parks since the Reform and Opening Policy in the late 1970s. In recent years, the number of industrial parks in China has increased year by year, ranking first in the world. As of 2019, the number of industrial parks in China has increased to 2543^2^. Industrial parks are the engines of economic growth in China, and the output value of industrial parks accounts for more than 60% of the country’s total industrial output values^3^. Meanwhile, industrial parks have consumed huge amounts of energy and resources, while emitting a variety of air pollutants. The development of green industrial parks has been emphasized in the national strategy of “Made in China 2025”^4^ and “Industrial Green Development Plan (2016–2020)”^5^ issued in 2015, implying that industrial parks had become an important area of air pollution control in China. Therefore, it is particularly urgent to evaluate the air pollutant emissions in industrial parks.

Establishment of emission inventory is the first step to grasp the emission characteristics and current status of air pollutant emissions in industrial parks, which will provide the basic emission information for policy-makers to formulate air pollution control strategies. At present, most studies on emission inventories focus on national, regional or provincial scales and specific industry. On the national scale, China’s first NMVOCs (Non-Methane Volatile Organic Compounds) emission inventory was established by Klimont et al.^6^. Konstantinos et al.^7^ developed a national annual SO_2_ emission inventory based on satellite data. On the regional level, Qi et al.^8^ developed a high-resolution air pollutant emission inventory for Beijing-Tianjin-Hebei region in 2013. Zheng et al.^9^ developed a highly resolved temporal and spatial Pearl River Delta regional emission inventory for the year 2006. In addition, there are more inventory studies focused on the provincial^10–12^ or city level^13–15^. It is worth noting that the previous results have shown that no matter which province or city, the contribution of industrial sources to air pollutants is particularly prominent, so further analysis of industrial sources is needed. However, the number of studies on air pollutant emissions from industrial parks is relatively small. For example, Gragava and Aggarwal^16^ established an air pollutant emission inventory for highly industrialized areas in southern India, but they did not analyze the source characteristics of specific industries. Wang et al.^17^ evaluated the environmental impacts of industrial symbiosis in an energy-intensive industrial park through the life cycle assessment method, in which the emission quantity of air pollutants was indirectly determined. Moreover, there were some studies on the air pollutant emission of chemical parks, but air pollutants are generally limited to VOCs^18–20^.

In the aspect of emission inventory in specific industry, previous studies were mainly concentrated on the iron and steel industry^21^, cement industry^22^, and coal-fired power industry^23^. Previous researches have laid the foundation for emission inventories for a single industry, but there were few studies on multiple industries. Industrial parks are gathering places for many different types of industries, so the establishment of park-level emission inventory is of great significance to industrial pollution study.

The previous studies of industrial parks mainly concentrated on the topics of energy consumption^24–26^, greenhouse gas mitigation^27–31^, green circular development^32,33^, and industrial symbiosis^34–36^. There were few studies on air pollutants in industrial parks, so it is urgent to analyze the air pollutant emissions in industrial parks. By determining the air pollutant emission inventory of these parks, the high-polluting industries in the industrial parks can be identified clearly. Furthermore, it can be reconfirmed that those industries with significant energy consumption also emit larger amounts of air pollutants. The findings of this study can certainly assist the decision-makers as to the priority for combating air pollution within their jurisdiction.

Due to the cluster of industries and the difficulty of data collection, the estimation of air pollutant emissions in industrial parks is challenging. To fill this gap, this study used 11 industrial parks as the research object and adopted a bottom-up approach to establish an air pollutant emission accounting framework for industrial parks. At the same time, we selected five indicators related to energy, air pollutants and industrial structure to scientifically classify these 11 industrial parks into three clusters in hierarchical cluster analysis method by SPSS software to discuss the emission characteristics of different types of industrial parks and make corresponding air pollutants reduction policy recommendations. On the basis, the emission reduction path of the industrial park and the emission reduction potential was further explored and estimated. Besides, we estimated the environmental benefits brought by different emission reduction paths. This is an attempt at a park-scale emission inventory that includes multiple industries and an exploration of the emission reduction management of different types of industrial parks.

## Methods

### Description of the study area

**Figure 1:**
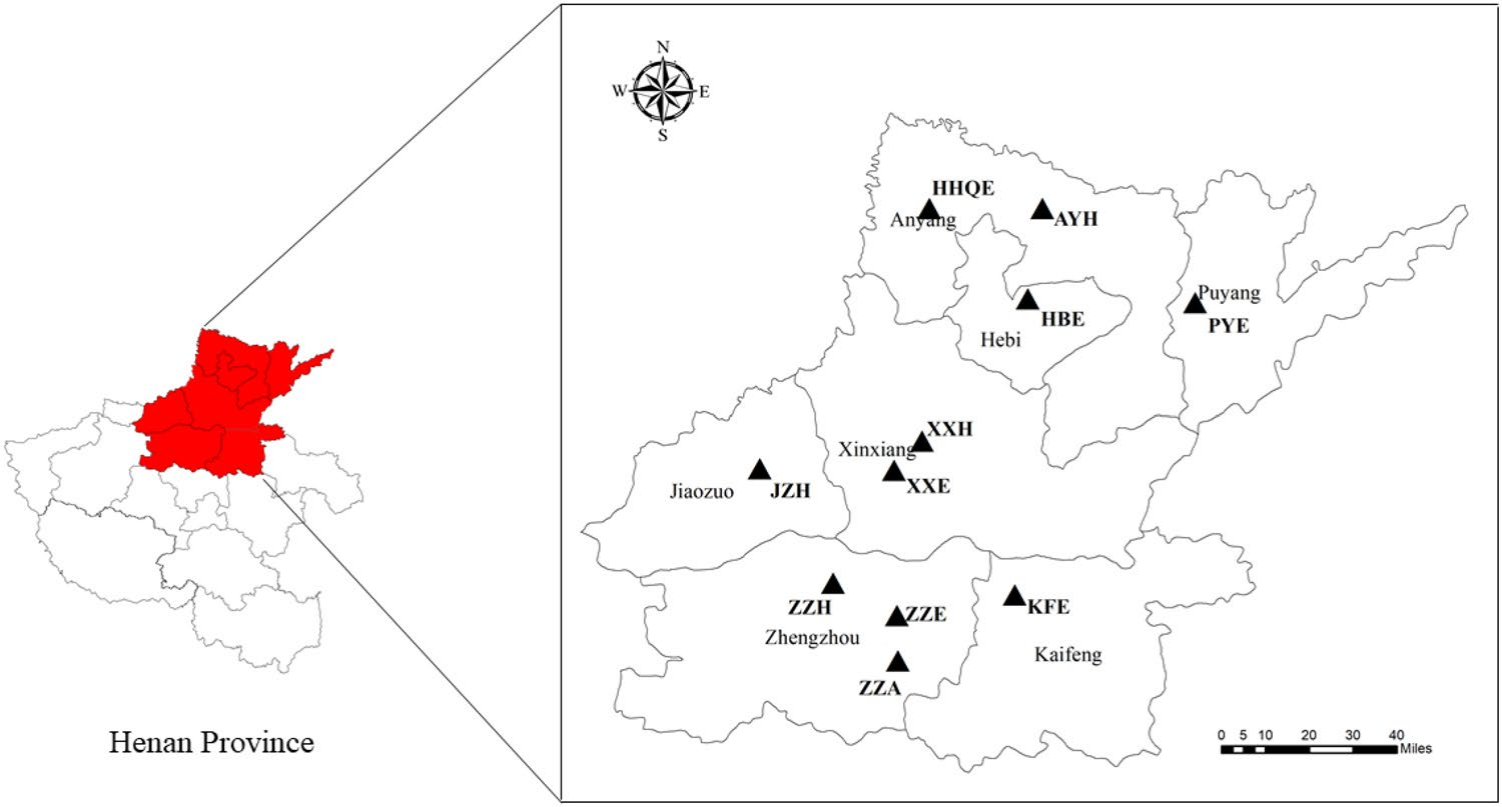
Location of 11 industrial parks in Henan Province. Map was created in ArcGIS Desktop v. 10.2 software. (http://www.esri.com/software/arcgis/arcgis-for-desktop/free-trial).

**Figure 2:**
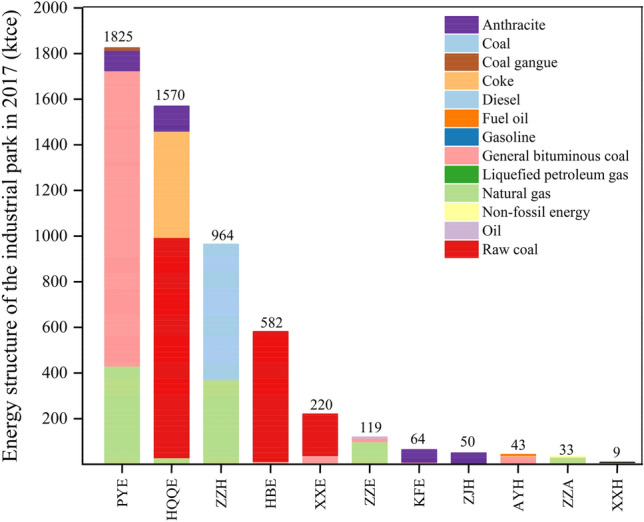
The energy consumption and structure of 11 industrial parks and proportion of energy consumption of each park to the overall energy usage in these industrial parks.

In this study, 11 industrial parks located in Henan Province were selected as the research objects, which were Puyang Economic and Technological Development Zone (PYE), Zhengzhou Economic and Technological Development Zone (ZZE), Kaifeng Economic and Technology Development Zone (KFE), Hongqiqu Economic and Technological Development Zone (HQQE), Hebi Economic and Technology Development Zone (HBE), Xinxiang Economic and Technology Development Zone (XXE), Zhengzhou High & New Technology Industrial Development Zone (ZZH), Anyang High & New Technology Industrial Development Zone (AYH), Xinxiang High-Tech Industrial Development Zone (XXH), Jiaozuo High & New Technology Industrial Development Zone (JZH), Zhengzhou Airport Economy Zone (ZZA). The geographic locations of the 11 industrial parks are shown in Fig. 1. According to China’s pollution survey, the total energy consumption of these 11 industrial parks was 5479 ktce in 2017. The energy consumption of each industrial park along with its contribution to the overall energy usage is shown in Fig. 2. Among these 11 parks, PYE had the highest energy consumption with 1825 ktce, of which bituminous coal accounts for 71% of the total energy consumption, followed by natural gas (23%); HQQE ranked the second with energy consumption of 1570 ktce, in which the consumption of raw coal accounts for the highest proportion (61%), followed by coke (29%); and ZZH ranks the third (964 ktce), in which coal consumption accounts for 61% and natural gas consumption accounts for 38%. In addition, the leading industries of each park and annual GDP of these industrial parks are shown in Table S1. The annual GDP of most industrial parks had little correlation with energy consumption. For example, the energy consumption of PYE was the highest, while the annual GDP was much lower. The reason is that the energy in PYE is mainly consumed by power plants, which contributes less economic output to the industrial park.

### Emission sources categorization

Emission sources categorization is the beginning of emission inventory accounting. The air pollutant emissions were calculated from the actual production perspective within the administrative boundaries of an industrial park. Due to the relatively high data requirements (such as waste treatment methods and volume) and small contribution on air pollutant emissions, fugitive sources are not included in this study. Based on the sources used for previous national and regional emission accounting and the structural characteristics of industrial parks, this study identified three sources for industrial park air pollutants emissions accounting, including power plants, industrial boilers, and industrial processes. Drawing on the National Economical Industry Classification^37^ and Technical Guideline for the Development of National Air Pollutant Emission Standards^38^, the emission sources of industrial process were divided into seven sectors, namely non-metallic mineral products industry (including cement, brick and tile industries), paper industry, textile printing and dyeing industry, ink printing industry, chemical industry, steel industry, and non-ferrous metal industry, as shown in Table S2.

### Emission estimation

According to the methods of compiling various inventory issued by the Ministry of Ecology and Environmental Protection of China^39–43^, this study used the bottom-up emission factor method and material balance method to estimate the emission inventory of 11 national industrial parks in Henan Province. Seven air pollutants, including SO_2_, NO_x_, CO, PM_10_, PM_2.5_, VOCs and NH_3_, were calculated in this study. Among them, SO_2_ emissions from power plants and industrial boilers were calculated by the material balance method due to the available sulfur content of the coal used for combustion. Emissions of SO_2_ from industrial processes and other pollutants from the three sources were calculated by the emission factor method. The calculation formula for pollutant emissions were as follows:

SO_2_ emissions from power plants and industrial boilers were estimated by the mass balance method^9^:

1 $$E_{s} = \sum\limits_{{i,}} {A_{{i,}} \times S_{{i,}} \times C \times (1 - \eta )} .$$

In the formula, *E*_*s*_ was the SO_2_ emission (t); *i* was the fuel type; *C* was the fuel-based coefficient (*C* = 16 when burning coal, *C* = 20 when burning oil, and *C* = 0.02 when burning natural gas^9^; *A* was the annual fuel consumption (t); *S* was the sulfur content of the fuel; and *η* was the desulfurization efficiency of SO_2_.

Other pollutants emission from power plants, industrial boilers and industrial processes were estimated by the emission factor method^9^:

2 $$E_{p} = \sum\limits_{{k,m}} {B_{k} \times EF_{{p,k,m}} \times (1 - \eta ) \times 10^{{ - 3}} }$$

In the formula, *E*_*p*_ was the total amount of pollutant emissions (t); *p* was the type of pollutant; *k* was the type of fuel or product;* m* was the control devices type; *B*_*k*_ was the activity level data (fuel consumption or product output, t); *EF*_*p,k,m*_ was emission factors (kg/t); and *η* was the removal efficiency of control measures. To better identify the spatial characteristics of pollutant emissions in 11 industrial parks, gridded emission inventory of industrial park was established at a resolution of 3 km × 3 km with the Geographic Information System technology. The emissions from power plants, industrial boilers and industrial enterprises are fixed point sources, and the geographic coordinates of enterprises or pollutant outlets were used to spatially allocate pollutant emissions. The results of various pollutants were distributed in a grid of 3 km × 3 km, and emissions from point sources were directly distributed to grid cells based on detailed longitude and latitude information.

### Activity data and emission factor

To establish the air pollutant emission inventory for industrial parks, we first classified the enterprises in each industrial park into different emission sources, and then collected the corresponding activity data and emission factor of individual enterprises. The activity level data of power plants mainly include energy type, energy consumption, boiler type, and end-of-pipe treatment technology. These data were from field surveys and government-organized pollution surveys. The power plant information of 11 industrial parks was shown in the Table S3. Among them, ZZH had two gas boilers with the largest installed capacity of 390 MW. The emission factor of a power plant was determined by its fuel type and combustion method. The emission factors were mainly derived from Ministry of Ecology and Environment and other literature, as shown in Table S4. Industrial boiler source was the enterprises that uses boilers for industrial production activities. The required activity level data include energy type, energy consumption, equipment type, and end-of-pipe treatment technology. The data of industrial boilers was from field surveys and pollution surveys organized by the government. There were 229 industrial boilers in 11 industrial parks, including 22 coal-fired boilers, 203 gas-fired boilers, 3 biomass boilers, and 1 oil-fired boiler. The source of emission factors of industrial boilers was the same as that of power plants, as shown in Table S5. Industrial processes sources refer to the sources of pollutants emitted into the atmosphere by industrial enterprises in industrial parks during product production and processing^44^. Activity level data mainly include the types of products produced by the company and the output of products and the installed pollutant removal facilities. These data were mainly derived from the industrial enterprise data of the additional pollution source survey in Henan Province in 2017, and corrected in conjunction with environmental statistics and field surveys. The source emission factors of the industrial process were mainly from the literature, as shown in Table S6.

### Classification of industrial parks using cluster analysis

The research objects were 11 industrial parks with different development scale, leading industries and energy consumption intensity. In order to better study the impact of industrial park types on air pollutant emissions, cluster analysis was used to classify the parks. Cluster analysis is an important means of data analysis in real-world scenarios^45^. This study used the Hierarchical cluster analysis method in SPSS (Statistical Package for Social Science) to classify the industrial parks. First, we selected 5 indicators to cluster the industrial parks based on literature^46^, which were the total industrial production value (x_1_), proportion of energy-intensive industries output value (x_2_), proportion of coal in the energy structure (x_3_), and energy consumption per unit of output value (x_4_) and pollution performance (x_5_). It was worth noting that the x_1_–x_4_ indicators were collected from field surveys or data reports, and x_5_ needs to be calculated. Seven kinds of air pollutants were used as industrial park classification indicators may cause inaccurate results of the park classification. Therefore, the environmental pollution performance was used here to represent the overall emission of air pollutants in the industrial park. Environmental performance refers to the measurable achievement of an organization’s environmental management system in controlling its environmental factors based on environmental policies, objectives and indicators^47^. In this study, the method proposed by Lucato et al.^48^ is adopted to evaluate the pollution performance of each industrial park. The principle of the evaluation method is to calculate the square of the radar graph consisting of the environmental indicators, so as to reflect the comprehensive situation of the industrial parks. By considering the requirements of sustainable economic development, the constraints of emission reduction policies and the current emission situation of industrial parks, this study selected seven air pollutant emissions indicators: the emission intensity of SO_2_, NO_x_, CO, PM_10_, PM_2.5_, VOCs, and NH_3_ (t/CNY, 1 CNY ≈ 0.15 USD in 2017). The pollution performance value is calculated by the Eq. (3) ^48^:

3 $$E_{i} = \left( {e_{{i1}} \times e_{{i2}} + e_{{i2}} \times e_{{i3}} + e_{{i3}} \times e_{{i4}} + \cdots + e_{{in}} \times e_{{i1}} } \right) \times \frac{{\sin \alpha }}{{2n}}$$

where *E* was the pollution performance, *i* was the industrial park, *e* was the emission intensity of seven air pollutants, *n* was the number of the evaluation indicator (*n* = 7), and *α* is the angle between two indicators in radar graph, which is equal to 360°/n. The value of pollution performance reflects the overall pollution status causing by different pollutants.

Then, the hierarchical cluster analysis method was used based on the above indicators selected to divide the parks.

### Emissions reduction path settings

In this section, we established the emission reduction paths for 11 industrial parks to quantitatively estimate the emission reduction potential of industrial parks (at the same level of energy consumption and product output in 2017). The first reduction path was energy adjustment measures (EAM), which was mainly focused on the replacement of energy intensive fuels and utilization of clean energy. Firstly, part of the coal-fired power plants was replaced by clean energy sources such as natural gas, which currently accounts for 10% of energy use. Secondly, in order to reduce the use of coal, all coal-fired boilers were replaced with gas-fired boilers. Thirdly, the end-of-pipe treatment technologies used by all industries remains the same as in 2017. In addition, according to the air pollution control policies issued by local governments, it was assumed that all coal-fired power plants under 30 MW in JZH and XXE will be closed. This assumption also applies to other measures. The second reduction path was promotion of end-of-pipe treatment technology measures (EPM), which mainly promotes the more advanced end-of-pipe treatment technology for each pollutant source. Currently, the 11 industrial parks promote the application of end-of-pipe treatment technologies. More advanced pipeline end treatment technology was installed in all pollution sources to remove pollutants, such as high-efficiency desulfurization devices and low-nitrogen combustion technologies and denitrification devices. In this measure, the technology with the highest removal efficiency was selected for each pollutant, and the detailed information of end-of-pipe treatment technology was shown in Table S7. The third measure was the comprehensive measures (CM), which combines EAM and EPM. Table 1 elaborates these three reduction paths. There was no special CO removal facility, and NH_3_ emissions mainly came from agricultural sources, so the air pollutants involved in the emission reduction path in this study only include SO_2_, NO_x_, PM_10_, PM_2.5_ and VOCs.

**Table 1:** Definitions of three reduction paths in this study.

	Clean energy alternative	End-of-pipe treatment technology promotion	Power plants	Industrial boilers	Industrial processes
Path 1—energy adjustment measures	✓	×	Natural gas replaces coal as a clean energy source, accounting for 10% of the energy used	Coal-fired industrial boilers are replaced by gas-fired boilers	Pollutants removal facilities will remain the same as in 2017
Path 2—promotion of end-of-pipe treatment technology measures	×	✓	Energy consumption is the same as in 2017	Energy consumption is the same as in 2017	Companies in the 11 parks all use more advanced end-of-pipe treatment technology
Path 3—comprehensive measures	✓	✓	Consistent with Path 1	Consistent with Path 1	Consistent with Path 2

### Environmental impact assessment

Air pollution was considered to be the greatest health risk related to environment^49^. To evaluate the environmental benefits brought to the industrial park under different emission reduction plans, this study selected five environmental impacts categories [acidification potential (AP), eutrophication potential (EP), particulate matter formation potential (PMFP), human toxicity potential (HTP) and respiratory inorganics (RI)] based on seven air pollutants and used quantitative methods to illustrate the environmental impact status of each industrial park. For the description of each environmental impact category, we first determined the listed environmental impact chemical categories from different literature sources and international methods^50–54^. Then, we used the air pollutant emissions calculated in this study and the equivalent factors of each environmental category collected in the literature to calculate the current environmental impact. The environmental impact categories and equivalent factors were shown in Table 2, and quantitative results of environmental impact reduction are calculated by formula (4):

4 $$EIA_{l} = \sum\limits_{j} {E_{s} \times EF_{j} }$$

${{EIA}}$ refers to the quantitative results of environmental impact reduction, *l* refers to the environmental impact category, j refers to the types of air pollutants; ${{E}}_{{ s}}$ refers to the emission reduction of air pollutants that determine the environmental impact, and ${{EF}}_{{j}}$ refers to the equivalent factor corresponding to the air pollutant.

**Table 2:** Selection of environmental impact categories.

Environmental impact	Parameter	Equivalence factor	Unit
AP	SO_2_	1	kg SO_2_eq
	NO_X_	0.7	
	NH_3_	1.88	
EP	NO_X_	0.13	kg PO4^3−^eq
	NH_3_	0.35	
PMF	PM_10_	1	kg PM_2.5_eq
HTP	SO_2_	0.31	kg 1,4-DCBeq
	PM_10_	1.2	
RI	PM_10_	0.536	kg PM_2.5_eq
	PM_2.5_	1	
	NO_X_	0.127	
	NH_3_	0.121	

## Results and discussion

### Source characteristics of overall emissions

**Figure 3:**
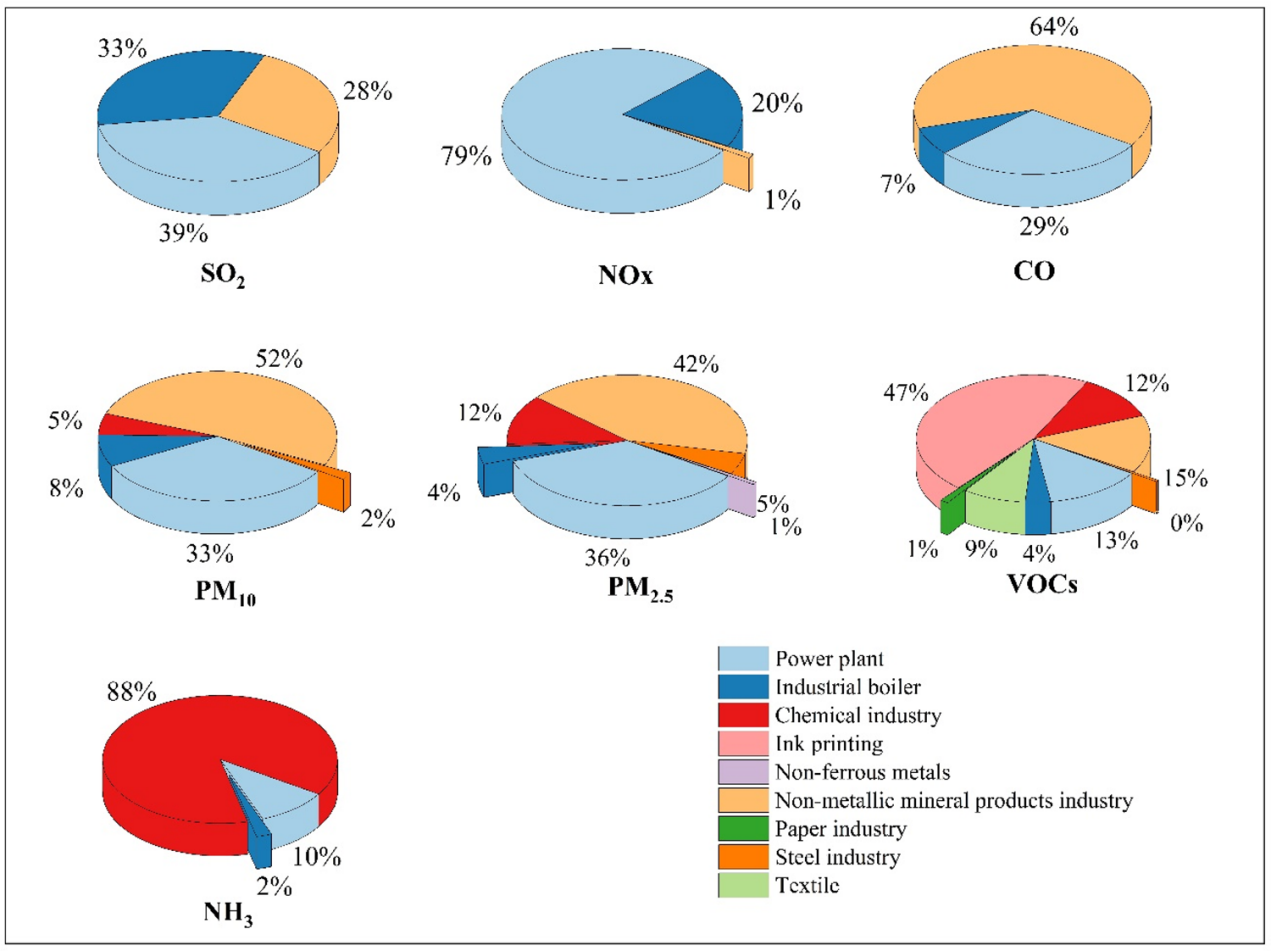
The contribution of nine emission sources to the overall emission of 7 pollutants in 11 industrial parks.

As shown in Table 3, the estimated emissions of SO_2_, NO_x_, CO, PM_10_, PM_2.5_, VOCs and NH_3_ in 11 industrial parks in 2017 were 11.2, 23.1, 30.8, 8.3, 3.5, 5.1, and 1.1 kt, respectively. The pollution sources of 11 parks were shown in Fig. S1. Due to the diversity of size, energy and industries structure of the parks, the results of different industrial parks varied greatly. The 2017-based air pollutant emission inventory of seven pollutants in industrial parks is summarized in Table 4. The contribution of each major category of nine emission sources to the emission of seven pollutants in 11 industrial parks was presented in Fig. 3. Power plants, industrial boilers, and non-metallic mineral products were the major contributors of the total SO_2_ emissions in 11 industrial parks and accounted for 39%, 33%, and 28% respectively. Compared with industrial boilers and industrial processes, power plants consumed huge amounts of energy. The amount of energy consumed by power plants was 1.6 times that of other sources, but SO_2_ emissions were only half of the other sources. The main reason is that the power plant has adopted strict control measures on SO_2_. According to China’s pollution survey, the power plants in these 11 industrial parks have installed desulfurization equipment at the end of 2017, with an average desulfurization efficiency of 80%. The large thermal power plants in 11 industrial parks had implemented ultra-low emission retrofits, which had almost lead to the maximum emission reduction. Therefore, the focus of reducing SO_2_ emissions in the future should be shifted to closing small thermal power plants and other control measures such as improving coal quality. Emission of power plants accounted for 79% of the total NO_x_ emissions, followed by industrial boilers with contribution of 20%. The burning of large amounts of coal and other fossil fuels in power plants and industrial boilers was the main reason for the high emissions of NO_x_. In 2017, the power plants in these industrial parks did not uniformly install more advanced nitrogen removal equipment such as Selective Catalytic Reduction with removal efficiency of 65%, which means that power plants have great potential to reduce NO_x_ emissions with more advanced nitrogen removal facilities. Take HQQE for example, NO_x_ emissions mainly come from power plants, with a contribution over 90%. Therefore, reducing coal usage as well as optimizing operating conditions (e.g., lower O_2_ level and combustion zone temperature) was the key to control NO_x_ in this park. Industrial processes are the main sources of CO, PM_10_, PM_2.5_, VOCs and NH_3_, among which different sub-sources have different contributions. Emissions from non-metallic mineral properties dominated the three pollutant emissions, totally contributing 64%, 52% and 42% to CO, PM_10_ and PM_2.5_, respectively. Industrial processes were the main contributors to VOCs. Among the sub-sources of industrial processes, ink printing had the largest VOCs emissions with a contribution of 47%. The second was the non-metallic mineral products industry, which contributed to 15% of the total VOCs emissions. NH_3_ was from the chemical industry, which accounts for 88% of the total emissions.

**Table 3:** The emissions of seven air pollutants in 11 parks in 2017 (unit: t).

Name	SO_2_	NO_x_	CO	PM_10_	PM_2.5_	VOCs	NH_3_
ZZE	308	157	1980	1450	472	239	2
KFE	1100	459	5720	520	150	1770	11
HQQE	2400	5190	10,400	580	390	479	34
HBE	894	2480	1600	1130	684	557	16
XXE	364	1160	514	427	112	90	5
PYE	4400	7670	4430	1800	767	479	996
ZZH	1070	5730	3400	787	338	1270	30
AYH	257	133	531	5	2	17	1
XXH	0.01	14	9	2	1	30	0.3
JZH	177	48	769	81	31	124	2
ZZA	223	69	1530	1510	516	53	4
Total	11,200	23,100	30,800	8290	3460	5090	1100

**Table 4:** Emissions of seven pollutants from major categories of emission sources in parks. (Unit: t).

Level 1	Level 2	SO_2_	NO_X_	CO	PM_10_	PM_2.5_	VOCs	NH_3_
Power plant		4740	18,200	9000	2750	1240	686	109
Industrial boiler		3520	4690	2150	661	148	180	24
Industrial process	Textile						446	
	Paper industry						59	
	Ink printing						238	
	Chemical industry				440	429	590	968
	Non-metallic mineral products industry	2930	262	19,700	4260	1440	740	
	Steel industry				156	184	24	
	Non-ferrous metals				21	24		
	Subtotal	2930	262	19,700	4880	2080	4230	968
Total emissions		11,200	23,100	30,800	8290	3460	5090	1100

### Spatial distribution of the emissions

**Figure 4:**
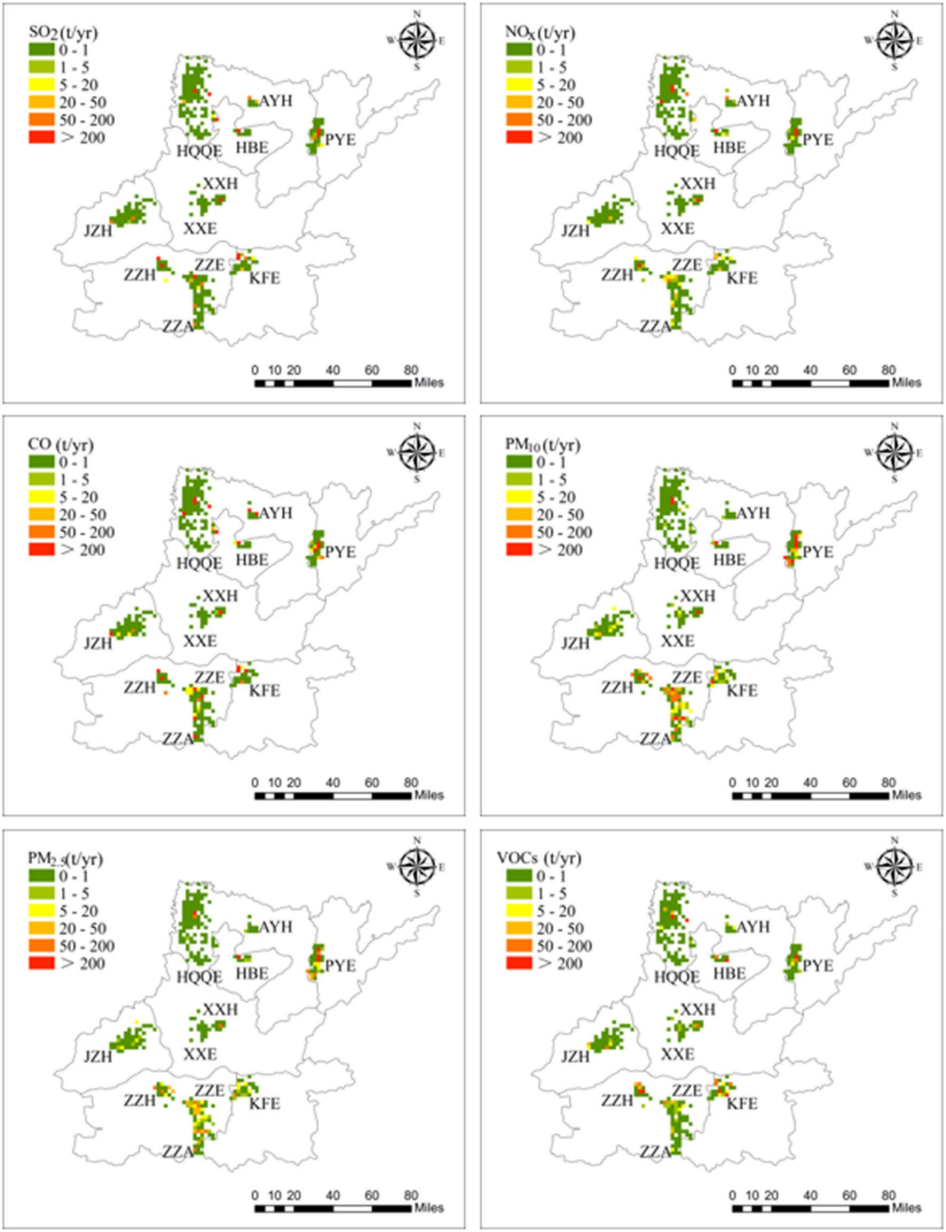
Gridded emissions of SO_2_, NO_X_, CO, PM_10_, PM_2.5_ and VOCs at 3 km × 3 km resolution in 11 industrial parks of 2017. Map was created in ArcGIS Desktop v.10.2 software. (http://www.esri.com/software/arcgis/arcgis-for-desktop/free-trial).

According to the longitude and latitude of each company, the pollutant emissions are allocated to the corresponding grid. The 3 km × 3 km spatial distribution of SO_2_, NO_x_, CO, PM_10_, PM_2.5_ and VOCs emissions was shown in Fig. 4, with seven pollutants emission inventory for each park illustrated in Fig. S1. Since NH_3_ emissions of industrial parks were small and the spatial distribution characteristics are not apparent, so the spatial distribution of NH_3_ was not considered. Comparing the spatial distribution of the six pollutants, the emissions of SO_2_ and NO_x_ were more concentrated, and the emissions in the high-value areas mainly came from industrial enterprises with power plants or industrial boilers. Especially for NO_x_, PYE and HQQE had more high-value areas than other parks. Due to the energy activities and industrial process emit a large amount of CO, no matter what type of park, there was generally a high-value area of CO emission. The emission high-value area of PM_10_ and PM_2.5_ were distributed in PYE, which was related to the emission source characteristics of PYE. The ZZE, ZZA, and ZZH located in the provincial capital city and PYE emit PM_10_ and PM_2.5_ emissions in a wide range of space. The main reason for this phenomenon was the large number and wide distribution of building materials enterprises in these parks. From a spatial point of view, VOCs was mainly distributed in ZZE and KFE, and other industrial parks emit less.

### Cluster analysis of industrial parks

**Figure 5:**
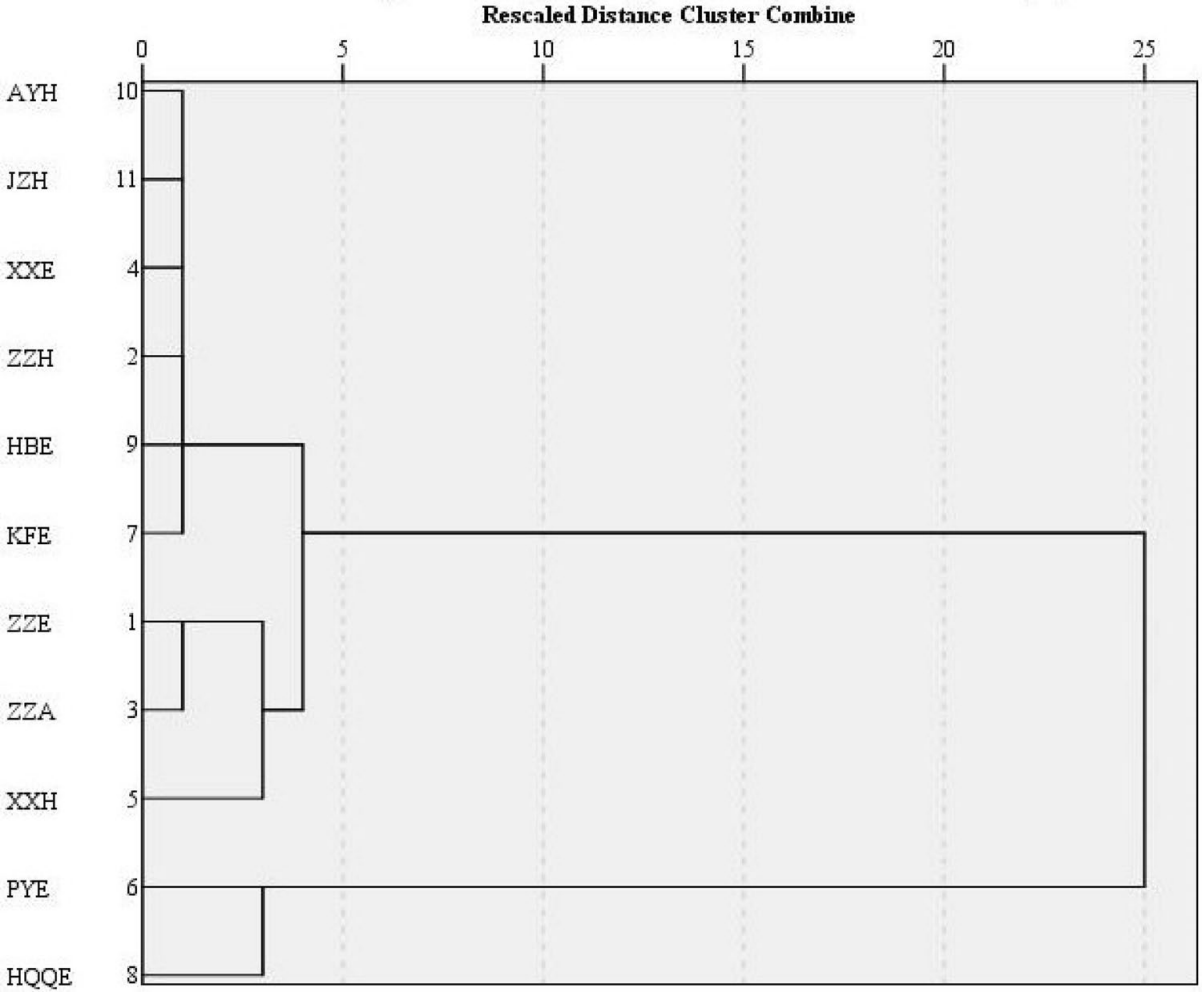
Dendrogram of industrial park by 5 indicators using hierarchical cluster analysis.

The detailed values of indicators x_1_–x_5_ were shown in Table S8. Among them, indexes x_1_–x_4_ were obtained by data collection, and x_5_ was obtained by calculation. It could be seen from the Table S8 that pollution performances (x_5_) of industrial parks were significantly different and extremely unbalanced with the range from 0.0001 to 294. To check whether the large difference between the upper and lower limits of the accounting range of this study was reasonable, this paper compared the results of previous studies^55^ (0.00012–1.27) and finds that our calculation results were reasonable. The detailed calculation of pollution performance for PYE was illustrated in Tables S9–S11 for reference. According to SPSS’s hierarchical cluster analysis, the industrial parks were divided into three groups (Table 5): cluster 1 includes PYE and HQQE; Cluster 2 includes ZZH, KFE, JZH, etc.; Cluster 3 includes ZZE, ZZA, and XXH. Dendrogram of industrial park was shown in Fig. 5. The 11 lines on the leftmost side of the ordinate represent the 11 industrial parks in this study. The lines from left to right continue to merge, and each merger represents a high degree of concentration. In other words, the merged line represents the same type of industrial park. In the distance of 0–5, it could be clearly seen that the 11 lines were merged into three independent lines, which represented the Cluster 1, Cluster 2 and Cluster 3. By comparing the campuses of each cluster, we found that each cluster has different characteristics. The total energy consumption of PYE and HQQE in cluster 1 was relatively high, and energy-intensive industries such as petroleum processing, chemical raw materials and ferrous or non-ferrous metal smelting and rolling processing were the leading industries, which the average output value of energy-intensive industries in the two industrial parks is 74.1%. In addition, their coal consumption accounts for a relatively high proportion of the energy structure, and their energy intensity industry was much higher than other clusters. At the same time, PYE and HQQE have the highest pollution performance values, which are 294 and 209, respectively. So, we marked the industrial parks in this cluster as “4Hs” parks (high energy intensity, high proportion of energy-intensive industries output value, high proportion of coal in energy mix and high pollution performance). Cluster 3 had three parks, ZZE, ZZA and XXH, which were all industrial parks dominated by emerging high-tech industries such as electronic information and automobile manufacturing, of which the average output value of energy-intensive industries is only 1.2%, much lower than other parks. And their energy intensity and coal consumption are much lower than other industrial parks. At the same time, their pollution performance value was also the lowest. Therefore, we marked them as “4Ls” parks (low energy intensity, low proportion of energy-intensive industries output value, low proportion of coal in energy mix and low pollution performance value). There were six parks in cluster 2, most of which were mixed parks, with both traditional industries and emerging industries. Their energy intensity, proportion of high energy-intensive industries, proportion of coal and pollution performance value were mostly between clusters 1 and 3. So, we marked them as “Mixed” parks.

**Table 5:** Clustering results of industrial park.

Category	Industrial parks	Characteristics
Cluster 1	PYE, HQQE	High energy intensity; high proportion of energy-intensive industries output value; high proportion of coal in energy mix; high pollution performance value
Cluster 2	KFE, AYH, JZH, ZZH, XXE, HBE	Medium energy intensity; medium proportion of energy-intensive industries output value; medium proportion of coal in energy mix; medium pollution performance value
Cluster 3	ZZE, ZZA, XXH	Low energy intensity; low proportion of energy-intensive industries output value, low proportion of coal in energy mix; low pollution performance value

### Air pollutant emission characteristics of different clustered parks

Due to the energy-intensive industrial structure, the “4Hs” parks had a high energy consumption and high emission of air pollutants. Their SO_2_, NO_x_, CO, PM_10_, PM_2.5_ and VOCs emissions accounting for 61%, 56%, 48%, 29%, 33%, 19% and 94% of the total emissions of the park respectively. It was worth mentioning that PYE’s NH_3_ emissions accounted for 90% of the total NH_3_ emissions, which was caused by several large synthetic ammonia companies in the park. The coal consumption of “4Hs” PYE and HQQE accounted for 62% of the total coal consumption of the 11 parks, which lead to high emissions of air pollutants and high pollution performance values. For “4Hs” parks, we must focus on promoting the development of clean energy, adjusting the industrial structure, and transforming energy-intensive industries to low-polluting industries. In addition, to achieve the purpose of reducing the pollution performance value of the park and promoting the green development of the park, the “4Hs” parks should also pay attention to the development of cleaner production and promote advanced end-of-pipe treatment measures. The emissions of SO_2_, NO_x_, CO, PM_10_, PM_2.5_, VOCs, and NH_3_ by the “Mixed” parks accounted for 34%, 43%, 41%, 36%, 38%, 75%, and 6% of the total emissions of the park respectively. Among them, the emissions of PM_10_, PM_2.5_ and VOCs in “Mixed” parks were the highest among the three-cluster parks. PM_10_ and PM_2.5_ was from energy activities, and production activities in cement industry were also one of the important sources. At present, “Mixed” parks had the problems of low application rate and backwardness of pollutant removal facilities. For example, the application rate of KFE’s VOCs removal equipment was only 36%, and the average removal efficiency was only 17%, while the removal efficiency of advanced VOCs removal facilities was as high as 80%. Therefore, “Mixed” parks should focus on promoting end-of-pipe treatment technologies. The “4Ls” Parks were dominated by emerging technology-intensive industries. Low energy intensity and high industrial production value are the main characteristics of this type of park. The emissions of “4Ls” parks were fewer than other types of parks, with the emissions of SO_2_, NO_x_, CO, PM_10_, PM_2.5_, VOCs, and NH_3_ accounting for 5%, 1%, 11%, 36%, 29%, 6%, 1% of the total emissions of the parks respectively. However, it was worth noting that the “4Ls” parks had higher PM_10_ and PM_2.5_ emissions. The reason was that ZZE and ZZA have great amount cement and other building materials industries, which consume more limestone and emits particulate matter. Therefore, while enjoying the economic contribution brought by emerging industries, the “4Ls” park should gradually eliminate high energy-consuming industries such as cement in the park and focus on the green development of the park.

### Estimation of emission reduction potential

**Figure 6:**
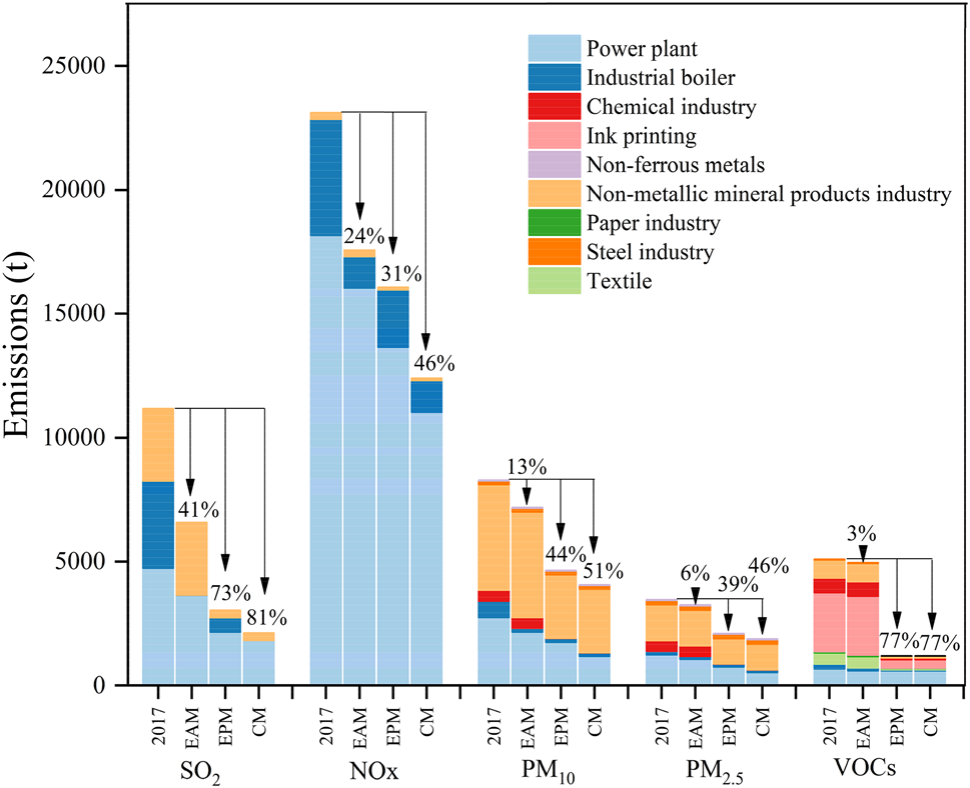
Sectoral emissions of major air pollutants from 11 parks in 2017 and three measures.

The estimated sectoral emissions of SO_2_, NO_x_, PM_10_, PM_2.5_, and VOCs for 2017 under three pathway measures were shown in Fig. 6. Both EAM and EPM could bring better emission reduction effects. Comparing the two measures of EAM and EPM, the emission reduction effect of EPM was obviously better than that of EAM. The reason was that the enterprises in these 11 industrial parks were still unfilled with the end-of-pipe treatment technologies. SO_2_, NO_x_, PM_10_, PM_2.5_ and VOCs emissions were reduced by 8.2 t (73%), 7.1 t (31%), 3.6 t (44%), 1.4 t (39%) and 4.0 t (77%) under the EPM with more advanced end-of-pipe treatment technologies, in which the emission reduction ratios are 1.8, 1.3, 3.3, 6.3 and 27.1 times as that of EAM respectively. It was noted that the emission reduction effect of VOCs was especially prominent in EPM. The main reason was that most of the existing enterprises in the industrial parks have not installed or installed VOCs removal equipment with low removal efficiencies, such as adsorption or external gas collecting hood-photolysis, which had installed more advanced removal equipment in EPM. The comprehensive measure combining these two measures had the largest emission reduction, and proves that there was great emission reduction potential of air pollutants in industrial parks, which was significant for the surrounding city or province to meet the national air quality standards. In the comprehensive measure, SO_2_, NO_x_, PM_10_, PM_2.5_, and VOCs were reduced by 9.1 kt (81%), 10.1 kt (46%), 4.2 kt (51%), 1.6 kt (46%), and 4.0 kt (77%), respectively. Among them, the emission reduction value of NO_x_ ranked first, and SO_2_ had the highest emission reduction ratio. For SO_2_ emission reduction, the priority was to reduce the coal consumption and improve the coal quality, such as replacing coal by natural gas and using low-sulfur clean coal. For NO_x_, it was necessary to implement ultra-low emission standards and optimize boiler operating conditions, e.g., increasing the oxygen-fuel equivalent ratio of the oxidant or reducing the furnace temperature^56^. At the same time, lowering the furnace temperature could also reduce CO emissions. For PM_10_, PM_2.5_ and VOCs reduction, it was necessary to strengthen the control of these pollutants in key industries such as cement, ink printing and chemical industries, and to improve the air pollution emission standards of these industries. For example, the current flue gas standard for PM_2.5_ of 30 mg/m^3^ in cement industrial^57^ should be lowered by 20% to meet the strict ambient air quality. To sum up, when industrial parks formulate air control policies, the promotion of advanced end-of-pipe treatment technologies should be a key measure to improve air quality at the national or urban regional scale^58, 59^. For the smooth promotion of this measure, the government could put forward some mandatory requirements. For example, the advanced desulfurization, denitrification, and dust removal equipment and technologies must be installed in the high-polluting enterprises which do not meet emission standards. However, due to the technological bottleneck effect, the reduction potential of air pollutants brought by end-of-pipe treatment measures is limited. Therefore, clean energy alternatives should become an important way to reduce pollutants and improve air quality in industrial parks in the long run.

### Environmental impact benefits

**Figure 7:**
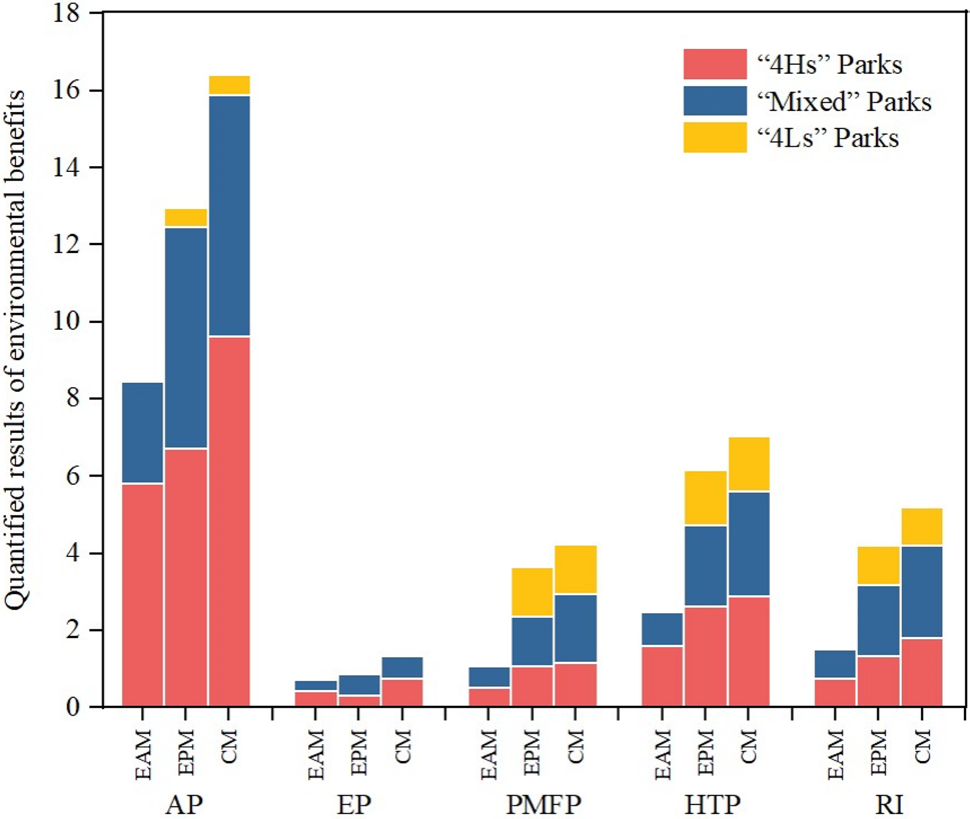
Five environmental benefits brought by three emission reduction measures. AP: acidification potential, kt SO_2_eq; EP: eutrophication potential, kt PO_4_^3−^eq; PMFP: particulate matter formation potential, kt PM_10_eq; HTP: human toxicity potential, kt 1-4-DCBeq; RI: respiratory inorganics, kt PM_2.5_ eq.

Figure 7 shown the results of the five environmental impact categories brought about by the three emission reduction measures, and detailed data were shown in Table S12. Due to the reduction of environmental impact was related to the emission reduction of pollutants, the environmental benefits of comprehensive measures are the greatest, which was reduced 16.4 kt SO_2_eq acidified gas emissions, 1.4 kt PO_4_^3−^eq eutrophication substances, 4.2 kt PM_10_eq atmospheric particulate emissions, 7.0 kt 1,4-DCEeq human toxic substances, and 5.2 kt PM_2.5_ eq breathing Inorganic. In recent years, particulate matter, particularly PM_2.5_, has been the primary pollutant in China^60^. PM exposure had a small but significant adverse effect on cardiovascular, respiratory, and, to a lesser extent, cerebrovascular diseases^61^. Regardless of the park clusters, the implementation of emission reduction measures can greatly reduce PMFP and RI, which not only improves the air quality of the park, but also benefits the health of surrounding residents.

### Uncertainty analysis

In this study, the uncertainty in the emission inventory of industrial parks was from two factors: the acquisition of activity level data and the selected emission factors. For the activity level data, the types of energy consumed of power plants and industrial boilers, energy consumption, and product output of industrial enterprises were all from environmental statistical yearbooks, government-organized pollution surveys and field surveys, which may differ from actual activity level. As to the selection of emission factors, due to the lack of localized emission factors for air pollutants, the emission factors in this study were mainly derived from the literature and standard values published by government agencies. In addition, the maintenance frequency and service time of pollutant removal facilities would have some influence on the pollutant removal efficiency.

## Conclusion

With the development of China’s industrialization, while the number of industrial parks is increasing year by year, we must urgently promote the development of green industrial parks. Understanding the emission characteristics of industrial parks so as to explore ways to reduce emissions is the foundation of any further air pollution controlling actions in industrial parks. This study established the park-level air pollutant emission inventory which includes fossil fuel emissions from power plants and industrial boilers as well as industrial processes and analyzed the emission characteristics of different clusters of industrial parks. Although different industrial parks may have different industrial structures and energy consumption levels, this inventory construction framework is applicable to industrial parks in other regions or countries. The pollution performance of 11 industrial parks was evaluated, and air control recommendations were provided for high-polluting industrial parks. Finally, the emission reduction potential and environmental benefits of 11 industrial parks under three emission reduction measures have been estimated. At the same time, these three emission reduction measures represent source reduction (closing small outdated coal-fired power plants), process control (clean energy use) and end-of-pipe treatment (promotion of end-of-pipe treatment technology) in the process of clean production pollution control. Therefore, the pollutant emission reduction measures proposed in this study were also applicable to other industrial parks, which could help them predict emission reduction potential and put forward relevant climate mitigation policy recommendations. The main conclusions of this study were as follows: (1) In 2017, the total emissions of SO_2_, NO_x_, CO, PM_10_, PM_2.5_, VOCs and NH_3_ in 11 industrial parks in Henan province in 2017 were 11.2 kt, 23.1 kt, 30.8 kt, 8.3 kt, 3.5 kt, 5.1 kt and 1.1 kt, respectively. Among the three sources of air pollutants in industrial parks, power plants are major contributors to SO_2_ and NO_x_. Industrial processes were major sources of CO, PM_10_, PM_2.5_, VOCs and NH_3_. (2) The 11 industrial parks in this study were divided into “4Hs” parks, “4Ls” parks and “Mixed” parks. “4Hs” and “Mixed” parks should be put in the priority in the air pollutant mitigation. They should not only focus on the transformation of traditional high-energy-consuming industries and the adjustment of energy structure, but also vigorously promote end-of-pipe treatment measures. The “4Ls” parks should gradually eliminate high energy-consuming industries such as cement in the park. (3) The measures of end-of-pipe treatment technologies and energy adjustment substitution can bring significant emission reduction potential. In comprehensive measure, SO_2_, NO_x_, PM_10_, PM_2.5_, and VOCs could reduce by 81%, 46%, 51%, 46%, and 77%, respectively. At the same time, the environmental impacts of AP, EP, PMFP, HTP, RI of 11 industrial parks were reduced by 16.4 kt SO_2_eq acidified gas emissions, 1.4 kt PO_4_^3−^eq eutrophication substances, 4.2 kt PM_10_eq atmospheric particulate emissions, 7.0 kt 1,4-DCEeq human toxic substances, and reduced 5.2 kt PM_2.5_ eq breathing inorganic. Furthermore, advanced end-of-pipe treatment technologies have better emission reduction potential than energy adjustment. (4) It was necessary to establish an air pollutant emission inventory for industrial parks, could provide important support for the government’s decision-making on air pollution control. Regarding the reduction of air pollutants in industrial parks, decision-makers can give priority to the promotion of end-of-pipe treatment measures and energy efficiency facilities, while gradually increasing the proportion of clean energy used in industrial parks. Moreover, it still requires huge efforts to tailor the emission reduction path for many industrial parks in various regions. We hope our study could be applied to the practice of air pollutant control in industrial parks and emission reduction management and could support the improvement of air quality in other industrial parks.

## Supplementary Information

Supplementary Information.

## Data Availability

The datasets generated during and/or analyzed during the current study are available from the corresponding author on reasonable request.
