# Supplementary material for: Uncovering the characteristics of air pollutants emission in industrial parks and analyzing emission reduction potential: case studies in Henan, China
Source: Sci Rep. 2021 Dec 9;11:23709. doi: 10.1038/s41598-021-03193-z (PMC8660784; doi:10.1038/s41598-021-03193-z)
Supplement: Supplementary file 1 — Supplementary Information. [file 41598_2021_3193_MOESM1_ESM.docx]

SUPPORTING INFORMATION

**Uncovering the characteristics of air pollutants emission in industrial parks and analyzing emission reduction potential: Case studies in Henan, China**

Gengyu Gao^1^, Shanshan Wang^2*^, Ruoyu Xue^1^, Donghui Liu^1^, He,Ren^1^, Ruiqin Zhang^2^

^1^ College of Chemistry, Zhengzhou University, Zhengzhou, 450001, China

^2^ School of Ecology and Environment, Zhengzhou University, Zhengzhou, 450001, China

**13 Pages, including:**

**Table S1** Information of 11 industrial parks.

**Table S2** Source classification of the power plant, Industrial boiler and industrial process.

**Table S3** Basic information table of boilers in power stations in national parks.

**Table S4** Emission factors for power plant sources.

**Table S5** Emission factors for Industrial boiler sources.

**Table S6** Emission factors for industrial process sources.

**Table S7** End-of-pipe treatment technology.

**Table S8** Pollution status of PYE.

**Table S9** Pollution indicators of PYE.

**Table S10** Calculation of PYE pollution index.

Table S11 Five environmental benefits under different emission reduction scenarios.

**Fig. S1** The major emission sources and composition of seven pollutants in 11 industrial parks.

| **Industrial park** | **Leading industry** | **2017 Gross industrial value (Billion CNY)** |
| --- | --- | --- |
| ZZE | Automobile and equipment manufacturing industry, modern logistics industry | 66.8 |
| KFE | Air separation equipment manufacturing, automobiles and parts | 8.4 |
| HQQE | Equipment manufacturing, auto parts processing industry | 15.1 |
| HBE | Chemical and building materials industry | 12.7 |
| XXE | Chemical fiber textile, automobile and auto parts industry | 11.2 |
| PYE | Chemical and equipment manufacturing | 13.5 |
| ZZH | Electronic information, new energy industry | 30.0 |
| AYH | Equipment manufacturing, electronic information industry | 3.5 |
| XXH | Electronic appliances and new biological medicine industry | 13.0 |
| JZH | Equipment manufacturing, new materials industry | 8.0 |
| ZZA | Electronic information industry, logistics industry | 70.0 |

**Table S1.** Information of 11 industrial parks

| **Level 1** | **Level 2** |
| --- | --- |
| Power plant |  |
| Industrial boiler |  |
| Industrial process | Non-metallic mineral products industry |
|  | Paper industry |
|  | Textile printing and dyeing industry |
|  | Ink printing industry |
|  | Chemical industry |
|  | Non-ferrous metals industry |
|  | Steel industry |

**Table S2.** Source classification of the power plants, Industrial boiler and industrial process.

| **Industrial Park** | **Power plant** | **Power station boiler type** | **Installed capacity (MW)** | **SO_2_removal technology** | **NOx removal technology** | **PM_10_/PM_2.5_ removal technology** |
| --- | --- | --- | --- | --- | --- | --- |
| JZH | P1 | Coal burning | 6 | Limestone-gypsum | SCR^b^ | Fabric filters |
|  | P2 | Coal burning | 12 | Limestone-gypsum | SCR | Fabric filters |
| PYE | P3 | Coal burning | 2×210 | Limestone-gypsum | SNCR^c^ | ESP^a^ combined with fabric filters |
| HBE | P4 | Coal burning | 2×135 | Limestone-gypsum | SCR | Wet scrubbers |
| HQQE | P5 | Natural gas | 9 | / | / | / |
|  | P6 | Natural gas | 6 | / | / | / |
|  | P7 | Natural gas | 6 | / | / | / |
|  | P8 | Natural gas | 9 | / | / | / |
|  | P9 | Coal burning | 2×350 | Limestone-gypsum | SCR | ESP combined with fabric filters |
| XXE | P10 | Coal burning | 1×18+1×12+1×25 | Double sodium-calcium | SNCR | ESP combined with fabric filters |
| ZZH | P11 | Natural gas | 2×390 | / | / | / |
|  |  | Coal burning | 2×135 | Limestone-gypsum | SCR | Fabric filters |

**Table S3.** Basic information of boilers in power stations inindustrial parks. ^a^Electrostatic precipitation；^b^Selective catalytic reduction; ^c^Selective non-catalytic reduction.

| **Sub-sector** | **SO_2_** | **NOx** | **CO** | **PM_10_** | **PM_2.5_** | **VOCs** | **NH_3_** |
| --- | --- | --- | --- | --- | --- | --- | --- |
| Pulverized coal furnace | 16×S | A | 2 | 46 | 12 | 0.15 | 0.02^g^ |
| Fluidized bed | 16×S | 1.5^c^ | 2.1^c^ | 1.54B^c^ | 0.45B^c^ | 0.15 | 0.02^g^ |
| Waste/Biomass | 2.67^a^ | 1.54^a^ | 3.6^e^ | 10.28^a,e^ | 5.88^a,e^ | 5.3^f^ | 0.04^b^ |
| Natural gas | 0.02S | 9.82^a^/1.66^a^ | 1.3 | 0.24 | 0.17 | 0.12^c^ | 0.05^g^ |

**Table S4.** Emission factors for power plant sources. (g/kg)

S is the sulfur content of the fuel, the value in this study is derived from the data provided by the power plant in the field survey.

A is related to installed capacity. If the installed capacity is smaller than100MW and there is no low nitrogen combustion boiler (LNB), the value of A is 10.5. If the installed capacity is bigger than or equal to 100MW and smaller than 300MW,at the same time there isno LNB, A is 8.85. If the installed capacity is larger than 300MW, at the same time there isno LNB, A is 5.85, and if there is LNB, A is 5.55.

B is the ash content in the fuel,the value source is the same as S.

^a^refers toCNE, 2011^1^, if power plant boiler without low nitrogen combustion boiler, the value is 9.82, if power plant boiler with LNB, the value is 1.66; ^b^refers to Pham, 2008^2^; ^c^refers to He et al., 2013^3^; ^d^refers to Huang et al., 2011^4^; ^e^refers to EPA, 2009^5^; ^f^refers to MEE, 2014^6^; ^g^refers to Yin, 2011^7^; unlabeled factorsrefers to Zheng et al.,2009^8^.

| **Sub-sector** | **SO_2_** | **NOx** | **CO** | **PM_10_** | **PM_2.5_** | **VOCs** | **NH_3_** |
| --- | --- | --- | --- | --- | --- | --- | --- |
| Layer burner | 16×S | 4 | 15 | 5.4 | 1.89 | 0.18 | 0.02^g^ |
| Pulverized coal furnace | 16×S | 4.72^a^ | 2^c^ | 3.51B^a^ | 0.22B^a^ | 0.18 | 0.02^g^ |
| Fluidized bed | 16×S | 7.5 | 2 | 28.08 | 5.04 | 0.18 | 0.02^g^ |
| Coke | 16×S | 4.8 | 6.6^c^ | 0.29^c^ | 0.144^c^ | 0.04^c^ | 0.02^g^ |
| Natural gas | 0.02×S | 2.09^d^ | 1.3 | 0.24 | 0.17 | 0.18 | 0.05^g^ |
| Diesel | 20×S | 9.62 | 0.6 | 0.5 | 0.5 | 0.15 | 0.13^g^ |

**Table S5.** Emission factors for Industrial boiler sources. (g/kg)

The meaning of B and S is referred to Table S3.

The source of emission factors is referred to Table S3.

| **Industrial process** | **classification** | **SO_2_** | **NOx** | **CO** | **PM_10_** | **PM_2.5_** | **VOCs** | **NH_3_** |
| --- | --- | --- | --- | --- | --- | --- | --- | --- |
| Non-metallic mineral products industry | Cement (grinding) |  |  |  | 8 | 2 |  |  |
|  | Cement (new dry process) | 4.8^c^ | 13.1^c^ | 40.2 | 8.05^e^ | 3.4^e^ | 0.18^d^ |  |
|  | Brick and tile | 0.6 | 0.05 | 4.04 | 0.13 | 0.04^a^ | 0.13^a^ |  |
|  | Refractories |  |  |  | 2.27^b^ | 1.63^b^ |  |  |
|  | Ceramics | 2.25 | 5 |  | 2.42 | 0.67 | 29.22 |  |
|  | Glass |  |  |  | 3.07^f^ | 2.94^f^ |  |  |
|  | Carbon |  |  |  | 1.6^f^ | 1.44^f^ |  |  |
| Non-ferrous metals | Electrolytic aluminum |  |  |  | 6.97^f^ | 5.2^f^ |  |  |
|  | Combined method |  |  |  | 56.4^f^ | 42.3^f^ |  |  |
|  | Bayer method |  |  |  | 12.4^f^ | 9.18^f^ |  |  |
| Chemical industry | Fertilizer |  |  |  | 2.12^f^ | 1.86^f^ |  | 2^n^ |
|  | Synthetic rubber |  |  |  |  |  | 7.17^f^ |  |
|  | Polypropylene |  |  |  |  |  | 3 |  |
|  | Viscose fiber |  |  |  |  |  | 14.5^f^ |  |
|  | Paint |  |  |  |  |  | 15^f^ |  |
|  | Synthetic ammonia | 3^h^ | 0.9^h^ | 142^n^ |  |  | 4.72^f^ | 2.1^n^ |
| Steel industry | Electric furnace steelmaking |  |  |  | 8.12^f^ | 6.02^f^ |  |  |
|  | Converter steelmaking |  |  |  | 14.68^f^ | 10.5^f^ |  |  |
|  | Hot rolled steelmaking |  |  |  |  |  | 0.3^f^ |  |
| Paper industry |  |  |  |  |  |  | 2.6^f^ |  |
| Ink printing |  |  |  |  |  |  | 750^f^ |  |
| Textile |  |  |  |  |  |  | 81.4^f^ |  |

**Table S6.** Emission factors for industrial process sources. (g/kg)

^a^is referred to EPA, 2009^5^; ^b^ is referred to Zhao et al.,2009^9^; ^c^ is referred to Lei et al.,2008^10^; ^d^ is referred to Bo et al.,2008^11^; ^e^ is referred to Lei et al., 2011^10^; ^f^ is referred to MEE, 2014^6^; ^h^ is referred to Zhao et al., 2012^12^; m is referred to Yin, 2011^7^;^n^ is referred to Zhao et al., 2012^12^; unlabeled factorsare referred to He, 2016^13^.

|  | **Decontamination methods** | **Decontamination efficiency (%)** |
| --- | --- | --- |
| SO_2_ | Sodium alkali desulphurization | 90^a^ |
|  | Limestone-gypsum | 80^e^ |
|  | Double sodium-calcium | 80^e^ |
|  | Flue gas circulating | 50^e^ |
| NOx | Selective catalytic reduction (SCR) | 65^a^ |
|  | Selective non-catalytic reduction (SNCR) | 30^a^ |
|  | SCR-SNCR | 50^a^ |
|  | LNB | 22^e^ |
| PM_10_/PM_2.5_ | Electric bag compound dust removal | 99.37^b/^99^c^ |
|  | Electrostatic precipitation | 96.7^b^/93^c^ |
|  | Fabric filters | 99.37^b^/99^c^ |
|  | Mechanical dust removal | 70^d^/10^d^ |
|  | Wet scrubbers | 79.57^b^/50^c^ |
| VOC_S_ | Adsorption-combustion | 80 |
|  | Precipitation separation | 30 |
|  | Low temperature plasma | 30 |
|  | Adsorption | 16 |
|  | External gas collecting hood-photolysis | 7 |

**Table S7.** End-of-pipe treatment technology.

^a^is referred to Xu, et al., 2017^14^; ^b^ and ^c^ is referred to MEE, 2014^6^; ^d^ is referred to Zhang ,2005^15^; ^e^ is referred to He, 2016^13^; unlabeled factors are referred to China's pollution source survey.

| Name | x_1_ | x_2_ | x_3_ | x_4_ | x_5_ |
| --- | --- | --- | --- | --- | --- |
| ZZE | 116.9 | 2.6 | 12.7 | 1.0 | 0.2 |
| ZZH | 30.0 | 21.4 | 58.6 | 33.9 | 22.2 |
| ZZA | 70.0 | 0.6 | 0.0 | 0.5 | 0.4 |
| XXE | 11.2 | 33.6 | 97.9 | 18.9 | 6.8 |
| XXH | 13.0 | 0.1 | 0.0 | 0.7 | 0.0 |
| PYE | 13.5 | 75.4 | 76.3 | 135.0 | 294.2 |
| KFE | 8.4 | 8.0 | 66.9 | 8.4 | 59.9 |
| HQQE | 15.1 | 72.8 | 56.3 | 129.1 | 209.2 |
| HBE | 12.7 | 12.5 | 98.6 | 46.0 | 37.5 |
| AYH | 3.5 | 10.2 | 71.2 | 11.6 | 5.7 |
| JZH | 8.0 | 7.5 | 85.9 | 6.8 | 1.2 |

**Table S8.** Index of Park Classification

Unit: x_1_:10^9^ CNY; X_2_: %; X_3_: %; X_4_: kt/109CNY.

.

| **SO_2_(t)** | **NOx(t)** | **CO (t)** | **PM_10_(t)** | **PM_2.5_(t)** | **VOCs(t)** | **NH_3_ (t)** | **GDP（Billion CNY）** |
| --- | --- | --- | --- | --- | --- | --- | --- |
| 4398.4 | 7667.7 | 4430 | 1794.5 | 766.8 | 478.8 | 995.9 | 135.4 |

**Table S9.** Pollution status of PYE.

*n* is the number of the evaluation indicator,n=7；α=360/n=360/7≈51.4°；sinα≈0.9

| **e_i1_** | **e_i2_** | **e_i3_** | **e_i4_** | **e_i5_** | **e_i6_** | **e_i7_** |
| --- | --- | --- | --- | --- | --- | --- |
| 32.5 | 56.6 | 32.7 | 13.3 | 5.7 | 3.5 | 7.4 |

Table S10. Pollution indicators of PYE

e_in_=Pollutant emissions / GDP

| **e_i1_×e_i2_** | **e_i2_×e_i3_** | **e_i3_×e_i4_** | **e_i4_×e_i5_** | **e_i5_×e_i6_** | **e_i6_×e_i7_** | **e_i7_×e_i1_** | **Total** |
| --- | --- | --- | --- | --- | --- | --- | --- |
| 1839.6 | 1852.8 | 433.6 | 75.1 | 20.0 | 26.0 | 238.9 | 4486.1 |

Table S11. Calculation of PYE pollution index.

$\text{E}_{\text{i}}\text{=}\left( \text{e}_{\text{i1}}\text{×}\text{e}_{\text{i2}}\text{+}\text{e}_{\text{i2}}\text{×}\text{e}_{\text{i3}}\text{+}\text{e}_{\text{i3}}\text{×}\text{e}_{\text{i4}}\text{+}\text{⋯}\text{+}\text{e}_{\text{in}}\text{×}\text{e}_{\text{i1}} \right)\text{×}\frac{\text{sinα}}{\text{2n}}$; *E_PYE_*=4486.1×(sinα/2n)=4486.1×（0.9/14）≈294.

| **Environmental impact** | **Park** | **EAM** | **EPM** | **CM** |
| --- | --- | --- | --- | --- |
| AP | “4Hs” | 5.8 | 6.7 | 9.6 |
|  | “Mixed” | 2.7 | 5.7 | 6.3 |
|  | “4Ls” | 0.1 | 0.5 | 0.5 |
|  | Total | 8.5 | 13.0 | 16.4 |
| EP | “4Hs” | 0.4 | 0.3 | 0.7 |
|  | “Mixed” | 0.3 | 0.6 | 0.6 |
|  | “4Ls” | 0.0 | 0.0 | 0.0 |
|  | Total | 0.7 | 0.9 | 1.4 |
| PMFP | “4Hs” | 0.5 | 1.1 | 1.2 |
|  | “Mixed” | 0.6 | 1.3 | 1.8 |
|  | “4Ls” | 0.0 | 1.3 | 1.3 |
|  | Total | 1.1 | 3.6 | 4.2 |
| HTP | “4Hs” | 1.6 | 2.6 | 2.9 |
|  | “Mixed” | 0.9 | 2.1 | 2.7 |
|  | “4Ls” | 0.0 | 1.4 | 1.4 |
|  | Total | 2.5 | 6.2 | 7.0 |
| RI | “4Hs” | 0.7 | 1.3 | 1.8 |
|  | “Mixed” | 0.8 | 1.9 | 2.4 |
|  | “4Ls” | 0.0 | 1.0 | 1.0 |
|  | Total | 1.5 | 4.2 | 5.2 |

Table S12. Five environmental benefits under different emission reduction scenarios.

Note: AP: acidification potential, kt SO_2_eq; EP: eutrophication potential, kt PO_4_^3-^eq; PMFP: particulate matter formation potential, kt PM_10_eq; HTTP: Human toxicity potential, kt 1-4-DCBeq; RI: respiratory inorganics, kt PM_2.5_ eq.





Figure S1. The major emission sources and composition of seven pollutants in 11 industrial parks.

# References

1. CNE, 2011. Handbook of Industrial Source Pollution Coefficient. China National Environmental Monitoring Centre. http://www.cnemc.cn/zzjj/jgsz/wrys/gzdt_wrys/201101/t20 110107_644997.shtml. (Accessed Sep 11, 2020).
2. Pham, T.B.T., 2008. Development of an inventory and temporal allocation profiles of emissions from power plants and industrial facilities in Thailand. Sci. Total Environ., 397, 103–⁠118. http://doi.org/10.1016/j.scitotenv.2008.01.066.
3. He, M., Wang, X.R., Han, L., 2013. Inventory and characteristics of atmospheric fixed pollution source emissions in Sichuan Province. Journal of Environmental Science., 33, 3127-3137. (In Chinese).
4. Huang, C., Chen, C.H., Li, L., Cheng, Z., Wang, H.L., Wang. Y.J., Huang, H.Y., Zhang, G.F., Chen, Y.R., 2011. Study on the characteristics of anthropogenic air pollutants emission in the Yangtze River Delta. Journal of Environmental Science., 31, 1858-⁠1871. (In Chinese).
5. EPA, 2009. AP–⁠42: Compilation of Air Emissions Factors [DB/OL]. United States Environmental Protection Agency. https://www.epa.gov/air-emissions-factors-and-quantification/ap-42-compilation-air-emissions-factors.
6. MEE, 2014. Technical Guidelines for Compilation of Air Pollutant Source Emission Inventory. Ministry of Ecology and Environment of the People's Republic of China. http://www.http://www.mee.gov.cn/ywdt/hjnews/201501/t20150113_294091.shtml.
7. Yin, S.S., 2011. Ammonia emission inventory of anthropogenic sources in the Pearl River Delta and its contribution to particulate matter formation, Environment Engineering, South China University of Technology, Guangdong. (In Chinese).
8. Zheng, J.Y., 2009. A highly resolved temporal and spatial air pollutant emission inventory for the Pearl River Delta region, China and its uncertainty assessment. Atmos. Environ.,43,5112-5122. https://doi.org/10.1016/j.atmosenv.2009.04.060.
9. Zhao, M., 2009. Evaluation of Air Pollutant Emission Reduction Effect during Beijing 2008 Olympics. Environmental Science and Engineering, Tsinghua University, Beijing. (In Chinese).
10. Lei, Y., 2008. Research on Emissions and Control of Particulate Matter and Key Chemical Components from China. [Ph.D. thesis] Tsinghua University, Beijing (In Chinese).
11. Bo, Y., Cai, H., Xie, S.D., 2008. Spatial and temporal variation of historical anthropogenic NMVOCs emission inventories in China. Atmos. Chem. Phys.,8,7297-7316. https://[www.atmos-chem-phys.net/8/7297/2008/](http://www.atmos-chem-phys.net/8/7297/2008/)
12. Zhao, Y., Nielsen, C.P., McElroy, M.B., Zhang, L., Zhang, J., 2012. CO emissions in China: Uncertainties and implications of improved energy efficiency and emission control. Atmos. Environ., 49, 103-113. https://doi.org/10.1016/j.atmosenv.201 1.12.015.
13. He, K.B., 2016. Technical Manual for Compilation of Urban Air Pollutant Emission Inventory. Tsinghua University Press, Beijing.
14. Xu, Y., Hu, J.L., Hao, H.K., Wang, D.X., Zhang, H.L., 2017.Current and future emissions of primary pollutants from coal-fired power plants in Shaanxi, China. Sci. Total Environ., 595, 505-514. https://doi.org/10.1016/j.scitotenv.2017.03.267.
15. Zhang, Q., 2005. Research on emission and simulation of fine particulate matter in China. [Ph.D. thesis] Tsinghua University, Beijing (In Chinese).
